# Commissioning and quality assurance for a respiratory training system based on audiovisual biofeedback

**DOI:** 10.1120/jacmp.v11i4.3262

**Published:** 2010-07-12

**Authors:** Guoqiang Cui, Siddharth Gopalan, Tokihiro Yamamoto, Jonathan Berger, Peter G. Maxim, Paul J. Keall

**Affiliations:** ^1^ Department of Radiation Oncology Stanford University Stanford CA USA; ^2^ Department of Music Stanford University Stanford CA USA

**Keywords:** respiratory training, audiovisual biofeedback, quality assurance, four‐dimensional computed tomography

## Abstract

A respiratory training system based on audiovisual biofeedback has been implemented at our institution. It is intended to improve patients' respiratory regularity during four‐dimensional (4D) computed tomography (CT) image acquisition. The purpose is to help eliminate the artifacts in 4D‐CT images caused by irregular breathing, as well as improve delivery efficiency during treatment, where respiratory irregularity is a concern. This article describes the commissioning and quality assurance (QA) procedures developed for this peripheral respiratory training system, the Stanford Respiratory Training (START) system. Using the Varian real‐time position management system for the respiratory signal input, the START software was commissioned and able to acquire sample respiratory traces, create a patient‐specific guiding waveform, and generate audiovisual signals for improving respiratory regularity. Routine QA tests that include hardware maintenance, visual guiding‐waveform creation, auditory sounds synchronization, and feedback assessment, have been developed for the START system. The QA procedures developed here for the START system could be easily adapted to other respiratory training systems based on audiovisual biofeedback.

PACS number: 87.56.Fc

## I. INTRODUCTION

Respiratory motion poses a significant challenge to the imaging of tumors in the thorax and upper abdomen, causing artifacts during three‐dimensional (3D) computed tomography (CT) image acquisition.^(^ ^1^ ^,^ ^2^ ^)^ These artifacts manifest themselves in the CT images in different ways, resulting in different errors and, consequently, diminishing the accuracy of diagnosis and radiation therapy during treatment planning^(^ ^3^ ^,^ ^4^ ^)^ and radiation delivery.^(^ ^5^ ^,^ ^6^ ^)^ One approach to reduce the motion artifacts is time‐resolved and ‐stamped CT imaging ‐ correlating respiratory motion in time with 3D‐CT image acquisition. This is often referred to as four‐dimensional (4D) CT.^(^ ^7^ ^,^ ^8^ ^)^ With 4D‐CT images, one can assess 3D tumor motion and directly incorporate that information into image reconstruction, thus reducing respiratory motion‐related artifacts. Even with 4D‐CT imaging, the current acquisition and reconstruction methods can still lead to significant artifacts^(^ ^9^ ^,^ ^10^ ^)^ (artifacts in one study measured >4mm in 90% of scans^(^ ^10^ ^)^) that can stem from several sources: (i) irregular breathing of a patient; (ii) reconstruction algorithm of CT images; and (iii) retrospective sorting of the reconstructed CT images into 3D volumetric image sets of different respiratory phases.

For instance, in axial cine mode, 4D‐CT images are acquired serially at each couch position. Retrospective sorting into different volumes is based on abdominal surface motion in the anterior‐posterior direction and temporal correlation to respiratory phases calculated by a breathing monitoring signal. The temporal coherence of retrospectively sorted 4D‐CT volumes, therefore, depends on the regularity of a patient's breathing pattern between couch positions. Unfortunately, ambiguities can arise when irregularities in breathing are present. The fundamental limiting factor here is the patient's irregular breathing pattern during acquisition. A clear link between respiratory irregularity and artifacts on 4D‐CT images has been demonstrated in several reports.^(^ ^11^ ^,^ ^12^ ^)^ It is also important to note that respiratory irregularity can cause artifacts for all imaging modalities, not just 4D‐CT imaging.^(^ ^13^ ^)^ Thus a device that can improve respiratory reproducibility of a patient during radiotherapy imaging is highly desirable.

Visual or audiovisual biofeedback has been demonstrated to reduce breathing variations in the mean cycle‐to‐cycle position, displacement, and period.^(^ ^14^ ^–^ ^16^ ^)^ A prototype audiovisual biofeedback device incorporating a patient‐specific guiding waveform has been reported previously by Venkat et al.^(^ ^17^ ^)^ Despite the initial promising results in improving the respiratory regularity with audiovisual coaching, residual cycle‐to‐cycle variations remain. An improved system, the Stanford Respiratory Training (START) system, has recently been implemented and tested at our institution. It features user‐friendly portable video goggles with full stereo audio and an interactive auditory function with low cognitive load for improving respiratory regularity. The system is able to prompt real‐time audiovisual biofeedback.

As outlined in the reports of the American Association of Physicists in Medicine (AAPM) Task Groups 40 and 76,^(^ ^18^ ^,^ ^19^ ^)^ whenever a new technology is being introduced into clinical practice, corresponding quality assurance (QA) measures should be taken. Although visual or audiovisual biofeedback systems are currently being used in several hospitals and clinics, no comprehensive QA procedures associated with them have been reported. This article aims to fill this gap and develop a comprehensive QA procedure for these systems based on the guidelines in the report of the AAPM Task Group 76.^(^ ^19^ ^)^ In this context, we describe the commissioning and QA procedures developed for the START system, which include hardware maintenance, software commissioning, interface with existing equipment, function assessment, and personnel training.

## II. MATERIALS AND METHODS

### A. System description and characteristics

A prototype of the system with preliminary evaluation has been reported previously.^(^ ^17^ ^)^ Significant improvements to that system include: more user‐friendly video goggles for patients' comfort, and an interactive piece of music for tracking patients' real‐time respiratory motion and providing auditory feedback. The improved system, or START system, consists of three main components: (i) a serial port communication protocol that interfaces the START system with an existing breathing monitoring device; (ii) comfortable, high‐resolution video goggles with full stereo audio that provides audiovisual signals to a patient; and (iii) in‐house developed software that generates audiovisual signals and graphic user interfaces.

**Figure 1:**
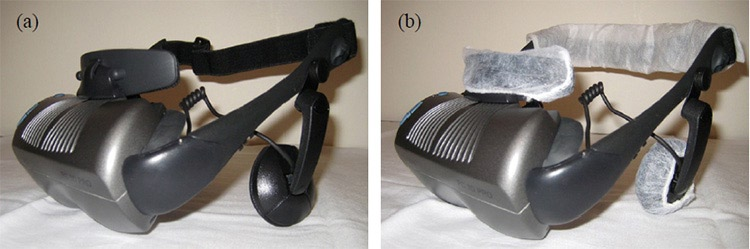
The headset of the video goggles, i‐glasses i3PC (a), and with hygiene covers (b) on its forehead support along with stereo phones and the head strap.

The START system has a dedicated control computer (Operating System: Windows XP Professional), to which the video goggles are connected and on which the START software runs. The system does not directly control any existing simulation or treatment equipment, but it does require a respiratory signal input. For this we use the Real‐time Position Management (RPM) system (version 1.7, Varian Medical Systems, Palo Alto, CA). While other respiratory signal inputs could be used, only the RPM has been used for patient studies. The real‐time respiratory signal is obtained by tracking the motion of an infrared (IR) marker block placed on the patient's abdomen by a tracking camera. It is output from the RPM system to the START system through a serial port connection, which can be enabled in standard RPM software. The video goggles are a portable computer monitor, i‐glasses i3PC (i‐O Display Systems, Sacramento, CA), whose relevant specifications are listed in Table 1, and whose headset is shown in Fig. 1(a).

**Table 1:** Specifications of i‐glasses i3PC video goggles.

Video Resolution (pixel)	800×600/LCD
Color Depth (bit)	24 (input)
Field of View (deg)	26 (diagonal)
Virtual Image Size (cm)	178 (as seen from 4 m)
Refresh Rate (Hz)	60 to 100
Weight (kg)	0.2
Cable Connector	VGA (DE‐15M)/Audio Jack (3.5 mm Male)/Power (9 V 800 mA)

The START software includes two pieces: a video piece and an audio piece. The video piece reads in a patient's real‐time respiratory signals from the RPM system at the beginning of simulation and creates a patient‐specific guiding waveform. The displacement of the guiding waveform is confined within an Inhale Limit line and an Exhale Limit line on the graphic interface for the patient. For guiding his or her subsequent breathing, the patient is instructed to limit the displacement of their breathing and, hence, that of the abdominal motion between these limits. Three parameters are used to characterize the guiding waveform and the respiratory trace: displacement, time period, and mean position. The mean position is defined as the average of the displacement in time from peak to trough.^(^ ^14^ ^,^ ^17^ ^)^ If the created guiding waveform is uncomfortable for the patient, it can be edited to offset the mean position, scale the magnitude of the displacement, or change the time period. An alternate strategy is to reacquire sample respiratory traces and create a new guiding waveform. But once deemed comfortable for the patient, it will be stored and kept the same for subsequent sessions.

The audio piece automatically reads in the data from the video piece. With improved function, the audio piece provides an interactive piece of music for tracking the patient's real‐time respiratory motion.^(^ ^20^ ^)^ Each piece of music is divided into one file containing the accompaniment and another containing the principal melodic materials of the file. The biofeedback is realized by the patient by adjusting his or her breathing pace or, equivalently, the real‐time variable tempo of the principal melodic line of the musical file, to synchronize with a separate accompaniment line or bass line whose tempo is predetermined by the time period of the patient‐specific guiding waveform. When the patient's breathing is regular and is at the desired tempo, the audible result sounds synchronous and harmonious.

**Figure 2:**
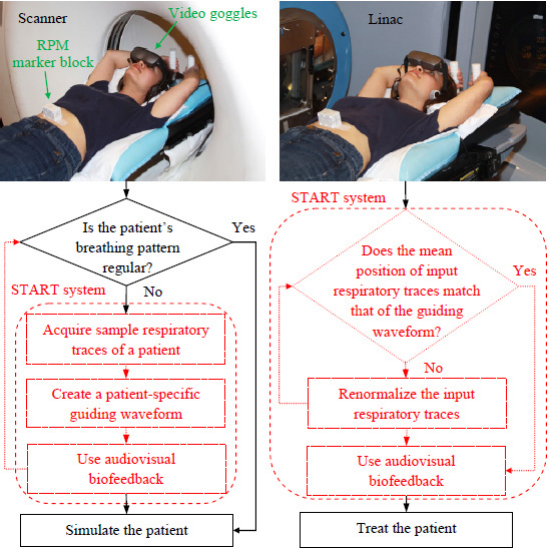
The general workflow diagrams of simulation (left) and treatment (right) using the START system. Shown at the top of the workflow is a volunteer in the simulation and treatment positions wearing the video goggles. The flowcharts with solid borders represent current clinical practice, while the flowcharts with dashed borders indicate added clinical practice associated with the START system. The RPM marker block is placed in the same location on the volunteer's abdomen for both sessions.

The general workflows of the START system for simulation and treatment are shown in Fig. 2. The flowcharts with solid borders represent current clinical practice without audiovisual biofeedback, while the flowcharts with dashed borders indicate added clinical practice associated with the START system. Our protocol is that if patients are simulated with the START system, they should be treated with the system. This, in turn, requires that the RPM marker block be placed in the same location on the patient's abdomen in simulation and treatment in order to get the required mean position of input respiratory signals to match that of the guiding waveform. However, during treatment, shifts can occur in the mean position of input respiratory signals due to potential changes in the relative position (or the distance) between the tracking camera and the RPM marker block, primarily caused by couch movements. A “Renormalize” function is designed to correct the shifts. This is realized by quickly acquiring four sample respiratory traces and recalculating the mean position of input respiratory signals. In case a patient feels uncomfortable with the START system, or the system fails to function properly, the fallback procedure is to disable the START system and revert to the current clinical practice of simulation and treatment without audiovisual biofeedback.

As the QA procedures related to the RPM system have been outlined in the report of the AAPM Task Group 76,^(^ ^19^ ^)^ we will only discuss the commissioning and QA procedures for the specific components developed for the START system. They could be easily adapted to other respiratory training systems based on audiovisual biofeedback.

### B. START system commissioning procedures

In order to commission the START system for clinical use many different operational aspects have been considered, such as: (i) video goggles setup and hygiene practice, (ii) START software commissioning, (iii) patient data storage, (iv) system backup, and (v) user guide development and personnel training. In the following sections, we focus on the commissioning and QA procedures for the first two operational aspects, since they are critical to the performance of the START system. The last three operational aspects are relatively straightforward, and corresponding results are given in Section III. A.3.

#### B.1 Video goggles setup and hygiene practice

The video goggles (i‐glasses i3PC) were set as the secondary monitor to the START system control computer. They were configured to display a read‐only graphic interface, from which the patient received instructions of what to expect and what to follow. Since the video goggles have direct contact with the patient's skin, a hygiene practice for the safety of the patient and the staff has been established. The detailed setup procedure and hygiene practice are given in Section III. A.1.

#### B.2 START software commissioning

The START software is the key component of the system and was the focus of the commissioning procedure. Four major tests were carried out for commissioning the START software: (i) the proper communication between the START system and the RPM system; (ii) the software's ability to create a patient‐specific guiding waveform and generate audiovisual signals for guiding the patient's subsequent breathing; (iii) the software's ability to detect an accidently used, non‐patient‐specific guiding waveform and, consequently, not to guide the patient's subsequent breathing; and (iv) the software's ability to renormalize input respiratory signals to get the required mean position to match that of the guiding waveform. We commissioned the START software based on these tests using the motor‐driven RPM marker block, which is commercially available (Varian Medical Systems, Palo Alto, CA). The results are given in Section III. A.2.

### C. Development of QA procedures for the START system

Frequent QA tests were developed for the START system that were categorized as daily (or prior to each simulation and treatment session, if used less frequently than daily) and monthly. The daily QA procedure included visual inspection of the hardware components and their connections, and functional tests of the auditory sounding and visual display of the video goggles. The monthly QA tests were designed to repeat the whole commissioning procedure for assuring the accuracy and maintaining the consistency of the clinical operation. The detailed QA steps are listed in Appendix A.

## III. RESULTS

### A. START system commissioning results

#### A.1 Video goggles setup and hygiene practice

The video goggles (i‐glasses i3PC) accept a VGA (800×600) video source input at 60 Hz and an audio source input (3.5 mm audio jack) with full stereo. Before first use of i‐glasses i3PC, the input VGA source needs to be set with 800×600 resolution at 60 Hz, and a minimum 16‐bit color resolution. These settings were configured under the “Display” category from the START system control computer.

**Figure 3:**
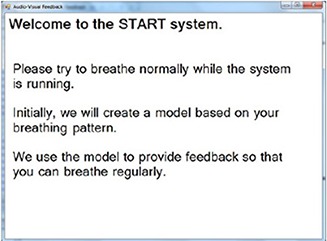
The initial “welcome” instruction on the video goggles screen.

Because the integrated cable provided by the factory for the video goggles is only about one meter long, an extension VGA/Audio/Power cable bundle (7.6 m for the simulation room and 15.2 m for the treatment room) was used to connect the video goggles with the START system control computer, which was placed outside the simulation and treatment rooms. Care must be taken when sending the extension cable bundle to the simulation and treatment rooms to avoid any change in room structure that could impact radiation shielding and increase radiation leakage. The video display and the auditory sounding of the video goggles were tested by playing a sample video with proper video resolution (800×600) and color resolution (16 bit). This sample video was also used for daily QA to ensure proper video display and comfortable volume. Figure 3 shows the initial “welcome” screen.

Disposable hygiene covers (see Fig. 1(b) were used to cover the forehead support, stereo phones and head strap where there is direct contact between the video goggles and the patient's skin. The goggles were thoroughly wiped with antiseptic before and after each use.

#### A.2 START software commissioning results

**Figure 4:**
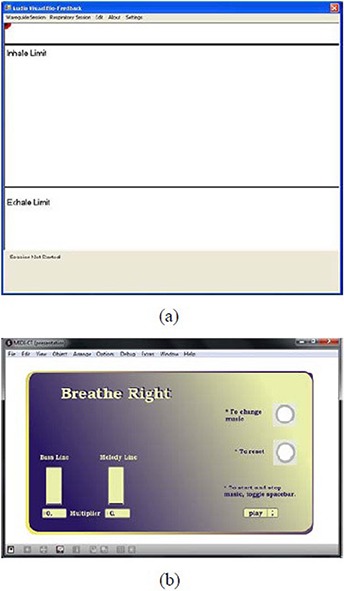
The starting GUI for the video piece of the START software (a); the Inhale Limit line and the Exhale Limit line are used to instruct patients to limit the displacement of their breathing and, hence, that of the abdominal motion. The starting GUI for the audio piece of the START software (b); the multiplier for the tempo of the bass line is predetermined by the time period of the patient‐specific guiding waveform. The multiplier for the tempo of the melody line is variable and negatively depends on the patient's real‐time breathing pace.

After the START software was launched, the initial graphic user interfaces (GUIs) for the video piece and the audio piece looked like what are shown in Figs. 4(a)) and (4b), respectively, from which the user operated the software and tested its functions.

**Figure 5:**
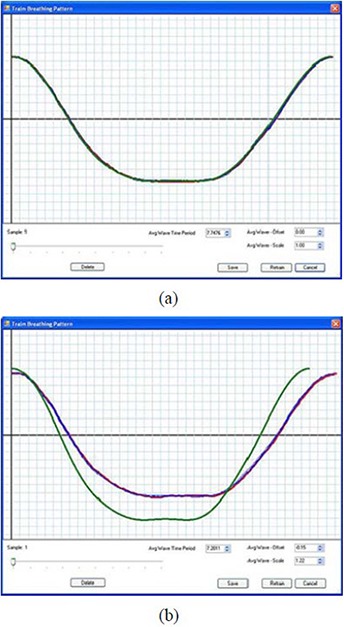
Sample respiratory traces of the motor‐driven RPM marker block (a); the average waveform is shown in green and overlaps with the sample respiratory traces. An edited average waveform (b) with the mean position offset negatively, the magnitude of the displacement scaled larger, and the time period reduced.

The communication between the START system and the RPM system was tested by acquiring sample respiratory traces (ten by default) from the RPM system. This step is defined as the Learn Waveform Session, as it is where the START system communicates with the existing breathing monitoring device and “learns” a patient's representative breathing pattern. Figure 5(a) shows the sample respiratory traces, which overlap with each other. This is because the motion of the motor‐driven phantom is regular and does not vary from cycle to cycle. By sliding the pointer along the sample track line, one can select each individual respiratory trace and may delete those that are deemed to be inappropriate (e.g. outlier). The average waveform is shown in green and also overlaps with the sample respiratory traces, from which the patient‐specific guiding waveform was calculated and constructed.^(^ ^17^ ^)^ If a patient feels uncomfortable with the guiding waveform, the average waveform can be fine tuned in order to offset the mean position, scale the magnitude of the displacement, or change the time period. Figure 5(b) shows an edited average waveform with the mean position offset negatively, the magnitude of the displacement scaled larger, and the time period reduced.

**Figure 6:**
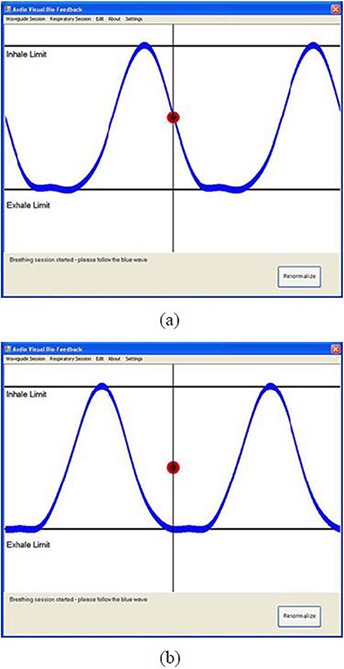
A snapshot (a) of the matched motions between the red ball and the blue guiding waveform. A snapshot (b) of the out‐of‐phase motions between the red ball and the blue guiding waveform.

A patient‐specific guiding waveform was constructed from the average waveform in Fig. 5(a). It was shown as a blue waveform on both the user and the patient's graphic interfaces, as shown in Fig. 6(a). The audiovisual feedback was tested by playing a session using the constructed guiding waveform to see if the patient's real‐time respiratory motion (represented by a red ball) was able to follow the patient‐specific guiding waveform and the audible result sounded synchronous and harmonious. This session is naturally called the Follow Guiding‐waveform Session. In practice, the red ball was moving up and down between the Inhale Limit line and the Exhale Limit line along the straight line in the middle of the figure, while the blue waveform was moving continuously from right to left across the screen. We confirmed that the red ball was able to follow the blue waveform in displacement, shape and time period. Figure 6(a) shows one snapshot of the matched motions. The audio function was automatically triggered at the beginning of the session, and the music sounded harmonious and synchronized. This was reflected by a constant multiplier for the tempo of the Melody Line in the bar indicator on the GUI for the audio piece.

When a non‐patient‐specific guiding waveform (e.g. one constructed from the modified average waveform in Fig. 5(b) was used to play a Follow Guiding‐waveform Session, the software was able to detect this and consequently did not guide the patient's subsequent breathing. The blue waveform in Fig. 6(b) is the correspondingly constructed guiding waveform. The audiovisual feedback was tested during a Follow Guiding‐waveform Session using this non‐patient‐specific guiding waveform. The red ball was found not to be able to follow the blue waveform in displacement, shape and time period. Its motion was out of phase with respect to the blue waveform as time went on, even though the red ball's motion and the blue waveform were in phase at the beginning of the session. Figure 6(b) shows one snapshot of the out‐of‐phase motions. The tempo of the melody line was not able to synchronize with that of the bass line, and the multiplier for the tempo of the Melody Line in the bar indicator varied drastically. The audible result sounded unsynchronized.

**Figure 7:**
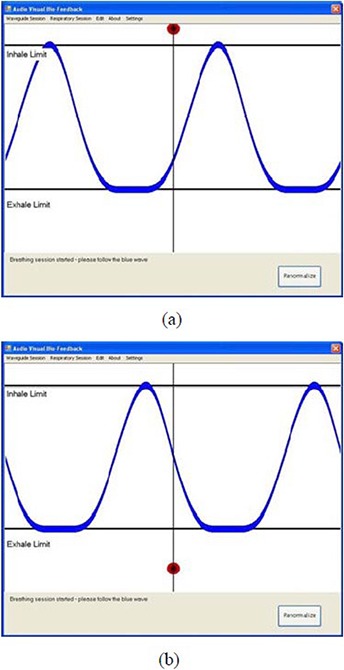
Snapshots of the out‐of‐limit displacement of the red ball's motion: (a) the displacement was out of the Inhale Limit line due to the up‐shifted RPM marker block; (b) the displacement was out of the Exhale Limit line due to the down‐shifted RPM marker block.

For the “Renormalize” function test, a change in the mean position of input respiratory traces was first introduced by shifting the vertical position of the RPM marker block up (or down) during a Follow Guiding‐waveform Session using the patient‐specific guiding waveform. This in turn led to the detected displacement of the real‐time respiratory motion to be out of the Inhale Limit line (or the Exhale Limit line), indicated by the red ball's motion, as shown in Fig. 7. By clicking the “Renormalize” button (shown in the bottom right corner of the figure), the shift in the mean position of input respiratory signals was able to shift back, resulting in a rematch of the red ball's motion and the blue guiding waveform, as in Fig. 6(a).

#### A.3 Data storage, system backup, user guide development, personnel training

Patient data, which includes the patient‐specific guiding waveform created in the beginning of the simulation and associated breathing data from subsequent sessions, are stored in a single location and can be accessed at all sessions. For the system backup, there is backup software and hardware redundancy. A User Guide for the START system has been developed. Staff training, including but not limited to the clinicians, physicists, and therapists, has been completed and can be requested as often as needed.

### B. START system QA testing results

The detailed QA steps are listed in Appendix A. The daily QA was straightforward, as it only involved visual inspection of the hardware components and a functional test of the video goggles. Since the monthly QA tests were designed to repeat the commissioning procedure, the testing results were the same as those shown in Figs. 5, 6 and 7.

### C. Initial volunteer and patient experience

**Figure 8:**
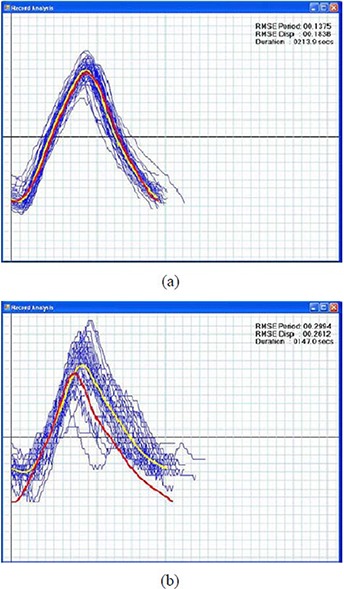
Example respiratory traces of a healthy volunteer (a) during training with individual breathing cycles (blue lines), a priori guiding waveform (red line) and post prior average waveform (yellow line). The RMSE in displacement and period are 0.2 cm and 0.1 s. The duration for the training is 3.6 min. Example respiratory traces of a lung‐cancer patient (b) during simulation. The RMSE in displacement and period are 0.3 cm and 0.3 s. The duration for the simulation is 2.5 min.

Upon the successful commissioning of the START system, we tested it with volunteers and patients having different breathing patterns and physiologic conditions. Each of the individuals participated in two sessions: the Learn Waveform Session and the Follow Guiding‐waveform Session. Shown in Figs. 8(a) and (b) are example respiratory traces with individual breathing cycles (blue lines) of a healthy volunteer during training and a lung‐cancer patient during simulation, respectively.

The red lines are a priori waveforms created during the beginning of the Follow Guiding‐waveform Session that were used for guiding the participants' subsequent breathing. The yellow lines are post priori average waveforms of the blue lines. The goal of the audiovisual biofeedback is to guide the participant's breathing using the a priori waveform such that, ideally, the post priori average waveform overlaps with the a priori waveform. To estimate the variations in the breathing traces of the Follow Guiding‐waveform Session, the root mean squared error (RMSE) in displacement (cm) and period (s) of breathing cycles were calculated and were shown in the top right corners of Figs. 8(a) and (b).

As shown in Fig. 8(a), the volunteer adapted to the START system very well, and the post prior average waveform almost perfectly overlaps with the a priori waveform. The patient was also able to quickly adjust her breathing pattern to adapt to the system after several breathing cycles, as shown in Fig. 8(b). Those blue lines in Fig. 8(b) that deviate substantially from the post priori average waveform indicate that the patient was able to adjust her breathing pace at the beginning of the simulation to follow the a priori waveform.

## IV. DISCUSSION

The intended applications of this device are for use during anatomic and functional imaging procedures such as 4D CT and positron emission tomography (PET) image acquisitions, as well as for treatment where respiratory irregularity is a concern. For 4D CT image acquisition and conventionally fractionated radiation delivery, sessions are typically several minutes long. The START system was able to help the patient improve respiratory regularity, as shown in Fig. 8(b). For 4D PET image acquisition, which typically takes several tens of minutes, it was found that there was little or even no improvement in the patient's respiratory regularity. This may be due to the fact that fatigue could affect concentration over longer time periods. For practical clinical use of the START system for PET imaging, audiovisual biofeedback with more intuitive and interactive components will be needed to help the patient concentrate. We also noted that most of the volunteers and patients were able to make their breathing regular using the visual guidance only. However, participants with a musical background reported that the auditory signal helped them keep their breathing rhythmic and made them relax as well. This may be partly due to the fact that the visual guiding waveform has a low cognitive load. We consider this important for future development of the audio biofeedback – for instance, by designing auditory signals with lower cognitive load.

## V. CONCLUSIONS

The START system was successfully commissioned and tested for clinical use at our institution. A detailed description of the commissioning procedure for the START software has been described. Results of these tests indicated that the system was able to prompt real‐time audiovisual biofeedback and help the patient improve respiratory regularity. Daily and monthly routine QA tests of this peripheral respiratory training system have been developed for assuring the accuracy and maintaining the consistency of the clinical operation. The QA procedure developed here for the START system could be easily adapted to other respiratory training systems based on audiovisual biofeedback.

## ACKNOWLEDGEMENTS

The authors gratefully acknowledge support from NIH/NCI R01 93626 and the Stanford BioX Interdisciplinary Initiatives Program (IIP). Sergey Povzner of Varian contributed significantly to the RPM real‐time output, and Raghu Venkat contributed significantly to the software development of early versions of the START system.

## Quality Assurance Worksheet for the Stanford Respiratory Training System

This document describes the quality assurance tests that should be performed after any software or hardware change to the Stanford Respiratory Training (START) system, or before each simulation and treatment with the START system.

***Daily QA or prior to each simulation and treatment if used less frequently than daily***

1. **Visual inspection of the hardware**
  Is there any visible damage to the START system control computer, extension cable bundle (VGA/Audio/Power), connectors, or the video goggles?
  □ Yes (Report)
  □ No
2. **Visual display and auditory sounding tests for the video goggles**
  Connect the video goggles with the START system control computer and play a sample video. Are the video displayed properly and the volume comfortable?
  □ Yes
  □ No (Report)
3. **START software graphic user interfaces**
  Start the audio piece of the START software first and then the video piece. Is the START system control computer showing the user interfaces as in Figs. 4(a) and 4(b), and the video goggles showing the patient interface with a “welcome” instruction as in Fig. 3?
  □ Yes
  □ No (Report)
  ***Monthly QA***
4. **Create a patient‐specific Learn Waveform Session**
  Create a new subject and acquire sample respiratory traces (ten by default) from the RPM system. Are the sample respiratory traces obtained successfully and saved?
  □ Yes
  □ No (Report)
5. **Play a Follow Guiding‐waveform Session using the patient‐specific guiding waveform**
  Play a Follow Guiding‐waveform Session using the patient‐specific guiding waveform. The audio piece should be toggled automatically and the music should sound synchronous and harmonious. Is the red ball tracing the blue guiding waveform in displacement, shape and time period?
  □ Yes
  □ No (Report)
6. **Create a non‐patient‐specific Learn Waveform Session**
  Create a new subject and acquire sample respiratory traces from the RPM system, then remove one sample trace and edit the average waveform time period, offset, scale, or any combination of them. Are all of these operations successful and the edited waveform saved?
  □ Yes
  □ No (Report)
7. **Play a Follow Guiding‐waveform Session using the non‐patient‐specific guiding waveform**
  Play a Follow Guiding‐waveform Session using the non‐patient‐specific guiding waveform. Is the red ball gradually moving out of phase with respect to the blue guiding waveform; and does the audible result sound unsynchronized?
  □ Yes
  □ No (Report)
8. **Renormalization function test**
  Play a Follow Guiding‐waveform Session using the patient‐specific guiding waveform and confirm the test passes. Then shift the vertical position of the motor‐driven RPM marker block up (or down). The displacement of the red ball should be out of the Inhale (Exhale) Limit line. Does the red ball trace the blue waveform again after clicking the “Renormalize” button?
  □ Yes
  □ No (Report)

Attestation

Physicist Name__________Signature__________Date______
